# On frail minds: addressing and assessing age-related neural decline and disease

**DOI:** 10.1007/s10433-018-0481-4

**Published:** 2018-06-13

**Authors:** David M. Lyreskog

**Affiliations:** 4TU Centre for Ethics and Technology, Universiteit Twente, Enschede, Netherlands

**Keywords:** Neurodegenerative disease, Frailty, Neural decline, Aging

## Abstract

The terminology surrounding frailty is used in clinical settings, and in research and development for identifying processes of, and patients in, age-related physical decline. However, a framework suitable for age-related neurodegenerative diseases needs to (1) adequately account for the effects that the processes of aging have on neural decline and disease, and (2) be helpful in identifying relevant groups of users and patients. This is becoming increasingly necessary due to emerging possibilities to detect, prevent, and treat age-related neural decline and disease. Based on a number of relevant criteria, I distinguish four groups of patients and users: robust, non-frail, pre-frail, and frail. With the four groups defined, ethical assessments can be made on an individual basis regarding which medical technologies are best suited for a person who risks, or suffers from, age-related neurodegenerative disease.

## Aging, frailty, and neurodegenerative disease

By the year 2050, the worldwide population of people aged 80 + will have quadrupled compared to now, and the number of people aged 100 + will have increased tenfold. At this rate, the portion of older adults is increasing faster than the general population. (UNFPA & Help Age International 2012). This increase in older populations is largely due to the development and distribution of effective health care. However, great challenges still lie ahead in health care for aging populations. The aging process is a complex and dynamic phenomenon. Although there are great variations in how aging expresses itself in different individuals, aging—after a certain point in life—is typically characterized by declines in function and capacity of homeostasis (Koga et al. 2011; Heikkinen, WHO Aging 1998; O’Neill 1997). This imbalance makes the human body susceptible to disease and increases the risk of organ failure. The neural networks of aging individuals are in no way excepted from the processes involved, as reduced capacity of homeostasis also contributes to neurodegeneration (Douglas and Dillin 2010).

When it comes to neural decline related to aging, there are great variations on individual level as well as on group level, where parameters such as lifestyle choices, environmental factors, and genetic disposition all have impact on the health of aging populations. For example, individuals who engage in intellectual activities are likely to develop a buffer against cognitive decline (Wilson et al. 2013; Hultsch et al. 1999), and physical activity seems to have a similar effect (Buchman et al. 2012; Lytle et al. 2004). Furthermore, environmental factors play a role, as pollution has been shown to speed up cognitive decline (Weuve et al. 2012; Power et al. 2011; Chen and Schwartz 2009). Genetic disposition is another factor, as it has effects on not only how sensitive an individual is to cognitive decline, but also on how the cognitive decline expresses itself (Deary et al. 2009). In these cases, *cognitive* decline can be understood as an effect of *neural* decline. As I will argue in what follows, I will mainly focus on the latter, understood as a reduced capacity of homeostasis and regeneration in neural networks.

The complexity of age-related decline has made it difficult to find a framework for understanding the processes involved. Over the last decade, the concept of frailty has been increasingly used in research and development (R&D) and in clinical contexts, framing processes involved in age-related decline. As the terminology has been developed, two kinds of frailty have been distinguished: physical frailty (Fried et al. 2001; Rockwood 2005), and cognitive frailty (Kelaiditi et al. 2013; Woods et al. 2013; Morley et al. 2015). Although these concepts are well developed, and helpful in many aspects of preventing and treating age-related decline, the terminology is insufficient when it comes to adequately accounting for the effects that the processes of aging have on neural decline and neural disease. To do so, it needs to go beyond what is commonly understood as strictly “physical” or “cognitive” ability (often measured though performance based methods), allow a more holistic view of frailty in aging, and account for pre-symptomatic conditions related to neurodegeneration. Furthermore, the existing terminologies are not helpful in identifying relevant groups of users and patients in the light of emerging technologies that may be able to detect, prevent, and treat neural decline and disease. Medical technologies and pharmaceuticals are currently under development that could change the way in which we view neurodegenerative disease and its precursors. To facilitate the development, the clinical use, and the ethical assessment of such technologies, the terminology surrounding frailty needs to be extended to include the context of neural decline and disease.

In this article, a suggestion in made on how and what to add to existing frailty terminology in order for it to accommodate for such applications in a way compatible with existing terminology. Ideally, an overarching framework, unifying the physical, cognitive, and neural aspects of frailty, should be constructed. Nonetheless, such a framework will arguably need to accommodate different types of frailty—including the one discussed in this paper.

In Sects. 2 and 3 of this article, I discuss the concepts of ‘physical frailty’ and ‘cognitive frailty’, and the terminology that has been developed around them. In Sect. 4 I argue that the existing terminology cannot be adequately applied to the context of neural decline and disease, and that an extension of the terminology is needed. Lastly, in Sect. 5, I suggest a way in which the terminology can be expanded so that it can account for age-related neural decline and disease, and facilitate technology development, clinical practice, and ethical assessment.

## Physical frailty

The concept of frailty, in the context of health and aging, was spelled out by Fried et al. (2001) in an attempt to provide criteria for how to define what was identified as a syndrome in aging populations (Rockwood et al. 1999; Speechley and Tinetti 1991; Winograd et al. 1991) and how to identify older adults who are at risk of physical decline. A person was to be considered as frail, if she met three or more of the following criteria:

- Weight loss (> 5% in last year);
- Exhaustion;
- Weakness (decreased grip strength);
- Slow walking speed (> 6–7 s for 15 feet);
- Decreased physical activity (males < 383 kilocalories; females < 270 kilocalories). (Fried et al. 2001, p. M148).

The idea was to establish objective (i.e., measurable and reliable) criteria for the syndrome in order to enable identification of older adults who are at “increased risk for future poor clinical outcomes, such as development of disability, dementia, falls, hospitalization, institutionalization or increased mortality” (The European Innovation Partnership on Active and Healthy Aging 2012). Over the last decade, the terminology surrounding frailty has been subject to critique, refinement and redefinition, but the criteria originally provided by Fried et al. (2001) are still often referred to, and used as a basis. (e.g., Rockwood 2005; O’Caoimh et al. 2014; Xue 2011).

Another line of the terminology that has had some success is the Frailty Index, and approach focusing on deficit accumulation of symptoms, impairments, abnormalities, and signs (Rockwood et al. 2015; Mitnitski et al. 2001; Goldstein et al. 2012). Rather than depending on the five criteria identified in the definition provided by Fried et al. (2001), a larger number of variables and indicators, including some cognitive deficits, are taken into consideration. The framework has been used to study the links between frailty, cognitive decline, and dementia (Godin et al. 2017; Montero-Odesso et al. 2016; Song et al. 2011).

The terminology surrounding frailty commonly identifies three distinct stages of the syndrome, and hence three groups of people: the ‘frail’, who show three or more symptoms of decline; the ‘pre-frail’, who show one or two symptoms; and the ‘robust’, who show no symptoms of decline (Xue 2011). Other versions of the terminology occur, however. Applying the terminology of frailty in the context of R&D, the project ‘Personalized ICT Supported Services for Independent Living and Active Aging’ (Persillaa) defined the pre-frail as “mild dysfunction in any of the three domains: cognition […] nutrition […] and physical (selected cut-off scores on a battery of physical assessments, adjusted for age and gender), and a Fried frailty score of 1 on 2.” (O’Caoimh et al. 2014). As of today, there is still no consensus regarding operational definitions for clinical purposes (Rodríguez-Mañas et al. 2013; Morley et al. 2013).

As we soon shall look at other forms of frailty, I will henceforth refer to the type of frailty discussed above as “physical frailty.” This is slightly misleading, as all types of frailty that are discussed here arguably are physical in some sense. Nonetheless, the terminology has developed in this direction over the last decade, and has been used to distinguish the physical aspects of frailty from cognitive frailty (e.g., Buchman et al. 2007; Kelaiditi et al. 2013).

## Cognitive frailty

While the aforementioned definitions of frailty have focused on what may be called physical function and decline, there are several studies suggesting that frailty is connected also to the decline of neural networks, increasing the risk of cognitive impairment, Alzheimer’s Disease (AD), and other dementias (Halil et al. 2015; Buchman et al. 2007; Kulmala et al. 2014; Gray et al. 2013). Despite these findings, the terminology describing the causes, effects, and expressions of frailty (i.e., physical frailty) has yet to be adapted. This terminological gap, and the concept’s failure to capture the variety of neural decline and diseases that commonly accompanies the aging process, has caused some concern. A consensus panel was therefore assembled by the International Academy on Nutrition and Aging, and the International Association of Gerontology and Geriatrics, in 2013 with the goal to “discuss current issues related to the relationship existing between frailty and cognition. Specific objectives of the meeting were: a) to summarize the existing literature in order to identify the papers that have examined whether frailty is capable to predict cognitive outcomes, b) to provide evidence showing links between frailty and cognition, c) to discuss and propose a first definition on cognitive frailty, d) to discuss and propose a list of screening tools and specific clinical and biological markers for identifying individuals at risk of physical disability and neurodegenerative disease, and e) discuss and propose potential preventive interventions about cognitive frailty.” (Kelaiditi et al. 2013, p. 727).

The panel concluded that there were indeed links between physical frailty and cognitive capability, but it also became clear that an operational definition of cognitive frailty was difficult to construct. The original definition proposed by the panel was the following:

[An] heterogeneous clinical manifestation characterized by the simultaneous presence of both physical frailty and cognitive impairment. In particular, the key factors defining such a condition include:

Presence of physical frailty and cognitive impairment. (CDR [Clinical Dementia Rating] = 0.5);

Exclusion of concurrent AD dementia or other dementias. (Kelaiditi et al. 2013, p. 731).

The term ‘cognitive frailty’ was thus meant to designate a condition in which the cognitive reserve is reduced *due to physical frailty*. This is also partly what differentiates it from an inverse of the cognitive reserve, which decreases the brain’s ability to resist damage (Woods et al. 2013). Also worth noting here is that the CDR score (0.5, on a scale 0–3.0) limits the scope to persons with very mild symptoms. Additionally, the following group distinctions were made:

1. Robust older individuals (i.e., no evidence of physical frailty) without cognitive problems (i.e., normal brain aging);
2. Physically frail older adults with normal cognitive functioning (as indicated by a Clinical Dementia Rating [CDR] equal to 0), including individuals with subjective memory complaints;
3. Older adults with no physical frailty but already exhibiting a cognitive impairment (CDR = 0.5);
4. Physically frail older adults with cognitive impairment (CDR = 0.5). (Kelaiditi et al. 2013, p. 731).

Of these groups, only group (4), the physically frail older adults with cognitive impairment, was defined as a condition of cognitive frailty. As the panel concluded that an operational definition is necessary, it made clear that further research is needed for such a definition to be established, as “available data are still preliminary and far to be conclusive” (Kelaiditi et al. 2013, p. 733). Indeed, the definition provided by the consensus panel has been criticized, although the intention has been applauded (Woods et al. 2013; Morley et al. 2015). Some issues that have been raised as critique are the exclusion of AD and other dementias (or other brain disturbances that could lead to dementia), and the scope of the definition due to its explicit limitation to people with CDR = 0.5 (Woods et al. 2013). Despite the difficulties in operationalizing the concept, screening tests for cognitive frailty have already been suggested (Morley et al. 2015). An expert panel at a consensus conference in 2015 defined cognitive frailty as “a reduced cognitive function (clinical dementia rating score 0.5) with the cognitive impairment being due to either physical or brain disease, or accelerated brain aging in the absence of evident brain disease” (Morley et al. 2015, p. 736). Additionally, it was yet again established that physical frailty has to be present for cognitive frailty to be present. Furthermore, the panel suggested that persons with physical frailty should also be screened for cognitive frailty, and vice versa. It was concluded that there are a number of instruments that could efficiently screen for cognitive frailty, and that all persons over 70 years of age should have their cognitive function tested at least once a year (Morley et al. 2015, pp. 736–37).

## Scrutinizing frailty

The terminology surrounding frailty needs to meet the following two criteria:

1. Adequately account for the effects that the processes of aging have on neural decline and disease.
2. Be helpful in identifying relevant groups of users and patients in the light of emerging technologies that may be able to detect, prevent, and treat neural decline and disease.

It is important that these criteria are met for several reasons, and they matter for different sets of stakeholders: for clinicians, an adequate terminology can be a reliable tool for identifying groups of patients that may be helped by a specific set of medical technologies. For people working in R&D of medical technologies, an adequate terminology facilitates the development of technologies tailored for certain groups of patients and users (i.e., non-patients) of health technology. Finally, the terminology is helpful for ethical assessment of medical technology development and application, as different values are at stake depending which technologies are used, and by whom.

Here, I argue that none of the conceptions of frailty presented in the previous section can meet criteria 1 and 2. The Fried frailty scale (Fried et al. 2001), and the conceptions and definitions strongly based on that scale (e.g., EIP on AHA 2012; Xue 2011; O’Caoimh et al. 2014), focus on physical frailty, and fail to adequately account for the effects that the processes of aging can have on neural decline and disease. Therefore, they cannot, as they stand today, meet criterion 1. Although these accounts are helpful in identifying groups that may run the risk of neural decline and disease (as physical and neural decline often coincide), they lack sensitivity to specific and pre-symptomatic biomarkers^1^ associated with neural decline and disease. Such sensitivity is necessary for early and effective interventions aimed at preventing and treating neural decline and disease, particularly in the light of emerging technologies that could efficiently detect relevant biomarkers. Therefore, the distinction between groups will be highly inaccurate, and the accounts based on the Fried frailty scale can thus not be said to fulfill criterion 2. Although some accounts mention cognitive aspects (e.g., O’Caoimh et al. 2014) as part of frailty, it is not clear how these accounts could be sufficiently helpful in identifying relevant patient groups.^2^ This focus on physical impairment, and negligence of neural and cognitive decline, has also been recognized by the scientific community (e.g., Kelaiditi et al. 2013).

The Frailty Index approach, although mainly focusing on physical aspects of frailty, does to a higher degree account for cognitive decline, and the relation between physical and cognitive aspects of frailty (e.g., Godin et al. 2017; Montero-Odesso et al. 2016; Song et al. 2011). Therefore, the approach holds the potential to meet criterion 1, given that the framework keeps developing. As it stands today, however, it focuses strongly on dementias, and little attention is given to other forms and/or expressions of age-related neurodegenerative conditions. Therefore, it can currently not meet criterion 1 to a sufficient degree. Furthermore, although the Frailty Index does a good job covering the connections between aging and frailty, it fails to capture specific pre-conditions and conditions of populations that risk neural decline and disease. There are populations that could benefit from medical intervention and monitoring, who have not necessarily accumulated a worrisome number of deficits according to the index (more on this below). Finally, and related, the comprehensiveness of the accumulative approach is at the same time its virtue and its vice: the extensive procedures required to determine type and degree of frailty in a single person would be costly, continuous, and time-consuming. It is unlikely that populations who are not community-dwelling, or do not already suffer from symptoms, would be evaluated: the costliness of continuous evaluations would presumably lead to that persons who are not experiencing symptoms (and therefore seek medical attention) and/or community-dwelling (and therefore under supervision) would not be evaluated at all. This includes large Non-frail and Pre-frail populations.

Taking a solely accumulative approach would therefore result in large un-diagnosed populations, who could have been helped by early and effective diagnosis. Although this does not necessarily affect the potential accuracy of such an approach negatively, it is a serious practical issue to take into consideration. As Cesari et al. (2013) have pointed out, different approaches to measuring frailty are good for different things. Perhaps a deficit accumulation approach such as the Frailty Index would be preferable in some populations, and less so in others.

Through recent developments, the terminology surrounding cognitive frailty has come closer to what is needed for the frailty terminology to account for age-related neural decline and disease. The focus on cognitive health, in addition to physical health, draws a clearer picture of how physical and cognitive processes interact and depend upon one another. The terminology also highlights the effects that aging has on cognitive functioning. Additionally, it has been recognized that early screening detection of biomarkers is not only increasingly possible, but also necessary to prevent severe cognitive impairment and dementia (Kelaiditi et al. 2013). However, when it comes to detecting, preventing, and treating neural decline and disease, the terminology is still insufficient to identify relevant groups of patients and users. First of all, the term “cognitive” is problematic, partly because it is unclear what functions it covers. Common definitions bring up memory, calculation, reasoning, or information processing ability (Brayne 2007; Morley et al. 2015). Such definitions are unclear in themselves (what does “reasoning” mean?) and often unjustifiably distinguished from supposedly separate psychological processes, such as emotion. This taints conceptions of neural decline and disease, as the neural systems that are damaged are plausibly not isolated to strictly cognitive functions (whatever they are taken to be).

Additionally, Woods et al. (2013) point out that it is unclear why the terminology should be restricted to persons with CDR = 0.5, and that the exclusion of persons with other brain disturbances (as suggested by Kelaiditi et al. 2013) might be misleading. I would like to take a step further and argue that these restrictions are highly problematic, if we want to identify persons who are at risk of, or suffer from, frailty in terms of neural decline and disease. Persons who have suffered from a stroke, for example, are at risk of increased dysfunction of the neural system, including dementias (Sun et al. 2014; Thiel et al. 2014; Rist et al. 2013). To not consider such persons frail is to miss a large group of people that could potentially be helped by emerging technologies. Persons scoring lower at CDR than 0.5 may also be frail (or, at the very least pre-frail), i.e., if they show biomarkers for neural decline or disease. For technologies aimed at *prevention* of neural decline and disease to be as effective as possible, individuals showing pre-symptomatic biomarkers ought to be covered by the terminology. For technologies aimed at *treatment* of neural decline and disease to be as effective as possible, individuals showing severe symptoms of dementia ought to be covered by the terminology. In this respect, the terminology of cognitive frailty fails to meet the two criteria required.

Another reason why the terminology of cognitive frailty is not apt is that it commits itself to persons suffering physical frailty. Although it is true that physical frailty often precedes age-related cognitive impairment and neurodegenerative disease, it is certainly not always the case. Kelaiditi and colleagues consider this in their group division, as they identify group (3) as “Older adults with no physical frailty but already exhibiting a cognitive impairment (CDR = 0.5)” (Kelaiditi et al. 2013, p. 731). It is not clear why the panel chose not to include this group of people, by adding coexistent physical frailty as a criterion for cognitive frailty. In any case, group (3) ought to be included in a terminology that seeks to identify persons risking neural decline and disease, since this group indeed risks neural decline and disease.^3^ Hence, the terminology of cognitive frailty fails to meet criterion 2, as it is not sufficiently helpful in identifying relevant groups of users and patients in the light of emerging technologies that may be able to detect, prevent, and treat neural decline and disease.

Yet another problematic issue is the focus on AD and other dementias. It is true that dementia, and AD dementia in particular, is common in aging populations. However, there are further severe and fatal neurodegenerative conditions that are not captured by the terminology. Parkinson’s disease, for example, grows increasingly common in aging populations (Levy 2007; Reeve et al. 2014; Cooper et al. 2015). The scope of the terminology needs to be wider to capture also other forms of age-related neural decline and disease.

Finally, a serious problem with the definitions available in the case of age-related neural decline and disease is the explicit commitment to older adults: both in the literature about physical frailty and in the literature about cognitive frailty, the definitions consistently and explicitly pick out older adults (see Fried et al. 2001; Kelaiditi et al. 2013). It is true that the conditions of neural decline and disease referred to are typically age-related. But as instruments for detecting biomarkers for neural decline and disease improve and get more precise, we will be able to detect the risk of decline and disease at early stages—before persons are reasonably to be considered “older” (late 60s and older). A call for explicitly excluding age from the frailty terminology has been made before, albeit for entirely different reasons. Markle‐Reid and Browne (2003) argued that including age in the concept of frailty suggests a negative and stereotypical view of aging. Although I do not want to argue their point of view here, I will argue that it is misleading to include age as such in the terminology. If that entails removing inexplicit negative stereotypes from the terminology as well, this is a pleasant side effect. In any case, it is not clear what reasons there could be to limit the scope to older populations. What is there to gain? I propose that age is removed from explicit definitions of frailty, and the definitions of the respective groups.

It can be concluded that, although cognitive frailty as a concept holds several qualities for theory and practice, it cannot be sufficient for the purposes sought after in this paper. Therefore, an expansion of frailty is necessary—one that is compatible with, yet goes beyond, existing accounts of physical and cognitive frailty.

## Re(de)fining frailty

I propose a terminology surrounding frailty, with regard to age-related neural decline and disease, that can (1) adequately account for the effects that the processes of aging have on neural decline and disease, and that can (2) be helpful in identifying relevant groups of users and patients in the light of emerging technologies that may be able to detect, prevent, and treat neural decline and disease. Notably, in doing this, I do not attempt to identify a syndrome. This is a common goal among many contemporary authors discussing frailty (e.g., Kelaiditi et al. 2013; Rockwood et al. 2005)—for good reasons. At this point, however, the goal is to provide a framework that is helpful in identifying and differentiating between groups of people that may be helped by emerging technologies for detecting, preventing, and treating age-related neural decline and disease.

**Fig. 1:**
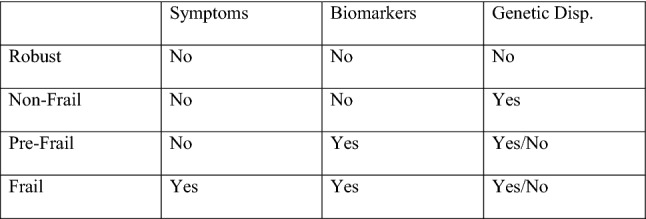
Tentative frailty groups, with regard to age-related neurodegenerative diseases

The structure of the terminology that I propose is based on the commonly used three-staged structure for differentiating groups in the literature on frailty (Xue 2011), containing the robust, the pre-frail, and the frail. However, I shall add a fourth group, the non-frail, to allow the terminology to account for the relevant differences and needs of the respective groups. I shall below define the groups and explicate how they relate to each other. The divisions are based on three variables, namely the presence of symptoms, biomarkers, and (currently non-detectable) genetic disposition (Fig. 1).

The presence of *symptoms* indicates damage to neural tissue, and is thus an important variable to consider when deciding which technologies to use. The detection of *biomarkers* for neural decline or disease is important in order to effectively prevent neural decline and disease from developing. This includes genetic as well as other biomarkers. In some cases, there are no symptoms or biomarkers indicating an increased risk for neural decline or disease. But there are types of neurodegenerative diseases that have hereditary abilities, to which we have yet to discover all reliable biomarkers. If the medical history of one’s family shows an abnormal occurrence of a certain neurodegenerative condition, one may want to take precautionary measures and use technologies that can detect biomarkers or symptoms, would they occur. Therefore, the variable of (currently non-detectable) *genetic disposition* is important when considering which technologies to use. That being said, there may be strong reasons to include more factors. More on this is below. Including these variables, the group division looks as follows:

The *robust* group consists of populations that show no symptoms of, and no biomarkers for, age-related neural disease or decline, according to instruments for detection. Nor do they run a significant risk of age-related neural disease or decline due to their genetic disposition. The individuals of this group are therefore not in any immediate need of technologies for preventing or treating neural decline. Nonetheless, they may benefit from using technologies for detection of biomarkers, for the purpose of monitoring their status.

The *non*-*frail* group consists of populations that show no symptoms of, and no biomarkers for, age-related neural disease or decline according to instruments for detection. However, the medical histories of their biological families indicate that they may run an increased risk of neural decline or disease. Some neurodegenerative diseases, such as Parkinson’s disease (Ibanez et al. 2004) and some forms of dementia (Loy et al. 2014; Edland et al. 1996) have been shown to have genetic precursors. These could be detected if we are able to identify the relevant biomarkers, but this is not the case for all forms of age-related neural decline and disease—at least not yet. As mentioned in the previous paragraph, more variables may be relevant to consider here. For instance, persons living under adverse living conditions such as high stress levels, or polluted environment, could arguably be considered non-frail based on such facts. For the sake of simplicity, I here let (currently non-detectable) genetic disposition serve as an example of what could group persons as ‘non-frail’, rather than stating that it is the only example. In any case, persons in the group ‘non-frail’ could benefit from using technologies to help detect anomalies, and that could prevent them from becoming pre-frail or frail, but generally not the same technologies as the two latter groups. For example, technologies for neural tissue regeneration or transplantation would not be helpful at a non-frail stage, as neural damage has not occurred.

The *pre*-*frail* group consists of populations that show no symptoms, but that show biomarkers for age-related neural decline or disease. They may or may not have a genetic disposition that increases the risk of decline or disease. This group contains individuals that show a significant risk of neural decline or disease according to detection technologies showing biomarkers for such conditions. Although the group does not show any symptoms of decline, they can benefit greatly from technologies that can prevent symptoms from emerging, and to keep their neural systems healthy. The pre-frail is, together with the frail, the group of people that may benefit the most from the technologies that are now emerging for detecting, preventing, and treating age-related neural decline and disease.

Finally, the *frail* group consists in populations that show symptoms of neural decline or disease. At this point, technologies for treating the conditions—to repair neural damage—may be implemented. The group ranges from persons showing few symptoms of decline, to persons in later stages of decline or disease that have an increased risk of mortality due to the severity of the damage done to neural tissue.

## Conclusion

I have suggested definitions of patient groups that I believe can (1) adequately account for the effects that the processes of aging have on neural decline and disease, and (2) be helpful in identifying relevant groups of users and patients in the light of emerging technologies that may be able to detect, prevent, and treat neural decline and disease. I have argued for a four-staged structure, including robust, non-frail, pre-frail, and frail populations, using three variables—symptoms, biomarkers, and (currently non-detectable) genetic disposition—as cornerstones. Tentatively, then, a definition of frailty in the context of age-related neural decline and disease could be formulated as follows: *a physical condition in which a person (a) shows symptoms of age*-*related neural decline or disease, (b) runs a significant risk of age*-*related neural decline or disease due to the presence of related biomarkers, or (c) may run an increased risk of age*-*related neural decline or disease due to (currently non*-*detectable) genetic disposition.* This account has a number of benefits compared to other existing accounts of frailty, making it more apt to the context of neural disease and decline:

- the terminology does not explicitly refer to the age of group members, widening the scope to include populations that are in need of early detection and prevention technologies;
- the terminology includes pre-symptomatic populations, an important feat when it comes to early and effective prevention of neural decline and disease;
- the terminology includes populations in later stages of decline and disease, something which is important when it comes to the emerging possibilities of treating severe neural damage;
- the terminology is applicable to technologies for detecting and identifying neural decline and disease, but also takes into consideration hereditary conditions with which we have yet to connect any specific and reliable biomarkers.

This extension of existing terminology can facilitate technology development, clinical practice, and ethical assessment. For clinicians, the terminology can facilitate the identification of groups of patients that may be helped by a certain set of medical technologies. For people working in R&D of medical technologies, the terminology could facilitate the development of technologies tailored for certain groups of patients and users (i.e., non-patients) of health technology. The terminology can also be helpful in ethical assessment of medical technology development and application, as different ethical issues are at stake depending on which technologies that are used, when, and by whom.

In moving toward a unified framework of frailty, including physical, cognitive, and neurological expressions of frailty, the terminology suggested in this paper is developed to be conceptually compatible with existing accounts of frailty. Further research needs to be conducted in how the suggested terminology should be applied in practice. This includes (1) further refinement of the terminology (2) development of effective tools for detection; (3) inquiry of the effects of those tools in practice, and (4) a feedback loop into continuous refinements and developments of terminology and technology. Due to many effective detection tools being still in their cradles, steps 2–4 are currently difficult to achieve, and could not be taken into consideration in this paper. Indeed, this paper seeks not to provide operational definitions, but rather to help lay a conceptual foundation of such definitions.

## Footnotes

1 The term “biomarker” here meaning “a characteristic that is objectively measured and evaluated as an indicator of normal biological processes, pathogenic processes, or pharmacologic responses to a therapeutic intervention” (Wagner et al. 2007).

2 Let me state clearly that none of the authors claimed that their terminologies could account for cases of neural decline and disease.

3 The concept of risk, and its relation to vulnerability, is highly relevant in the context of frailty. How we conceive of frail populations, e.g., as being vulnerable to and/or at risk of suffering from a set of conditions, rather than actually suffering from any of them, can and should arguably affect the structure of decision-making processes. The limited scope of this paper unfortunately prevents a fair exploration of related issues.
